# Sensory Assessment by Consumers of Traditional and Potentially Probiotic Green Spanish-Style Table Olives

**DOI:** 10.3389/fnut.2018.00053

**Published:** 2018-06-26

**Authors:** Antonio López-López, José M. Moreno-Baquero, Francisco Rodríguez-Gómez, Pedro García-García, Antonio Garrido-Fernández

**Affiliations:** Food Biotechnology Deparment, Instituto de la Grasa (Consejo Superior de Investigaciones Científicas), Sevilla, Spain

**Keywords:** green Spanish-style table olives, potentially probiotic olives, consumer test, principal component analysis, predictive biplot, canonical variate analysis

## Abstract

This work presents the sensory characterization by consumers of traditionally and potentially probiotic green Spanish-style table olives. To this aim, green Manzanilla olives from the same lot were debittered, washed, and brined in the same way; then, one sub-lot was allowed fermenting spontaneously while another one was inoculated with a putative probiotic bacterium (*Lactobacillus pentosus* TOMC-LAB2). After fermentation, the olives from both sub-lots were packed with fresh brine to reach 5.5 g/100 mL NaCl and 0.6 g lactic acid/100 mL in the equilibrium. The stabilized olives were then subjected to sensory evaluation by 200 consumers, and the results were analyzed by ANOVA and multivariate statistical techniques. In a first approach, consumers perceived the spontaneously fermented olives as similar to the potentially probiotic product. However, a biplot based on Canonical Variate Analysis (CVA) showed differences between them in the Salty and *Overall score*. When data from the consumer test were assessed by PLS analysis, regardless of the fermentation system, *Overall score*, and *Buying predisposition* were significantly driven by Appearance, Odor, Salty (negatively), Hardness, and Crispness.

## Introduction

Diverse foods are used as carriers for delivering probiotics to humans. Until recently, the term probiotic was almost exclusively associated with dairy products. However, Chiu et al. (1) showed that *Pediococcus pentosaceus* and *Lactobacillus plantarum* isolated from pickled vegetables were able to inhibit the *Salmonella* invasion in mice. Atrend toward the use of other food systems like vegetables as carriers (2–4) is nowadays evident. A first approach can be the addition of the potentially probiotic organism. *L. plantarum* and *Lactobacillus paracasei* survived in artichokes for at least 90 days, and the anchoring to the product improved their survival through the gastrointestinal tract; particularly *L. paracasei* IMPC2.1 was recovered from stools (5). *L. paracasei-*enriched artichokes had a favorable effect on constipated patients (6) and *L. paracasei LMGP22043* incorporated into artichokes transiently colonized the gut, antagonized with *Escherichia coli* and *Clostridium* spp. and increased the genetic diversity of lactic acid bacteria (LAB) (7). The adhesion of Lactobacilli and bifidobacteria onto ripe table olives with the aim of formulating a new probiotic food has also been reported (8).

But the lactic acid fermentation of vegetables is an ancient tradition from all around the world and would be an attractive mechanism for incorporating the appropriate bacterium into their final products. The use of LAB with specific characteristics as starter cultures is a common practice in diverse plant materials. Its application may lead to faster acidification and other technological, nutritional, health, or safety advantages as well as to produce a favorable influence on the organoleptic properties (9). The use of the probiotic *L. plantarum* L4 and *Leuconostoc mesenteroides* LMG 7954 as starter cultures for cabbage head fermentations led to products which could be considered as probiotic [alive cell counts >6 log_10_ CFU/g product; Verganovim (10)]. A starter culture of *L. plantarum* optimized the lactic acid fermentation of York cabbage and produced a ~5 log_10_ CFU/mL increment in bacterial growth. The population level persisted for longer than 15 days' storage in a cold room (4°C); therefore, inoculation can be used for developing potentially probiotic products (4). However, the matrix may play an important role in the probiotic development and efficacy (2). Due to the peculiar characteristics of table olives (11), the use of an adapted starter culture could result essential for its implantation. Mourad and Nour-Eddine (12) were the first to make an *in vitro* a *L. plantarum* preselection from olive microbiota based on probiotic criteria. Bevilacqua et al. (13) chosen potential multifunctional starter cultures from Bella di Cerignola table olives. Hurtado et al. (14) reviewed the use of LAB starters and outlined the possibility of using table olives as probiotics. Bautista-Gallego et al. (15) made a screening of LAB, mainly *Lactobacillus pentosus*, from wild Spanish-cultivar fermented table olives to be used later as probiotic starters. Argyri et al. (16) have selected from Greek cultivars several strains of *L. plantarum* and *L. paracasei* with similar properties than *Lactobacillus casei S*hirota and *Lactobacillus rhamnosus*. Botta et al. (17) investigated the microbiota of the Italian cultivar Nocellara Etnea to identify new probiotics LAB with the same objective, suggesting *L. plantarum* S11T3E as an interesting candidate. Peres et al. (18) also characterized the potential probiotic features of strains of LAB from Galega fermentations, finding 10 strains belonging to *L. plantarum* and *Lactobacillus paraplantarum* who had probiotic value. Recently, Guantario et al. (19) found one strain of *L. pentosus* and *Lactobacillus coryniformis* who were able to out-compete foodborne pathogens for cell adhesion and were promising stater candidates for manufacturing table olives with probiotic added value.

In any case, the presence of the strain in the brine does not assure its intake. It is essential to study their incorporation onto the olives since only these are ingested. De Bellis et al. (20) used *L. paracasei* IMPC2.1 to control the green Spanish-style fermentation and colonize the olive surface. A biofilm formation on glass and fruits during green Gordal (21) and Manzanilla (22) table olive fermentation has been reported. The imposition of the probiotic strain on olives has been demonstrated to be highly dependent on its characteristics, circumstance that confirms the importance of the matrix and a proper starter. In green olives cv. Halkidiki, inoculated with *L. pentosus* B281 and *L. plantarum* 282, the first strain was best adapted to the fermentation environment and survived in high number in both low (8% NaCl) and high (10% NaCl) brines while the second did not survive at 10% NaCl; besides, when inoculated on co-culture, only *L. pentosus* B281 was recovered at a high number (>90%) from the olive fruits (23). Similar results were also observed using heat shocked green olives where both strains dominated over the indigenous LAB, but *L. pentosus* B281showed also higher proportions of recovery in the 10% NaCl brine and dominate in the case of co-culture (24). As observed, an adequate starter can provide a considerable LAB load on fermented olives. An exhaustive report on the diverse strains of LAB and yeasts proposed as starter cultures can be found elsewhere (25). As a result, several reports have emphasized the role of table olives as adequate carriers for delivering probiotic bacteria to humans (26, 27), particularly when the LAB strains were previously isolated and characterized from olive microbiota (25). Among the challenges mentioned by Champagne and Gardner (28) for the probiotic production are the determination, in the product, of the appropriate cell population, and the effect that the potentially probiotic starters had on the sensory properties of the fermented products. In inoculated fermented green Spanish-style olives from the diverse cultivars and countries, the sensory characteristics of the fermented product were comparable to those of the traditional lactic acid fermentation (23, 29), but when using heat socked green olives, the sensory assessment of products showed higher preference for the olives from probiotic inoculated fermentations (24). The analysis of consumer preferences for table olives, in the case of Albanian urban consumers, is an interesting approach to disclose the motivation regarding driving olive consumption, mainly with the interest of improving the local offer (30). However, the comparative response of consumers against the traditional and probiotic origin packaged table olives has been scarcely studied and still represents an attractive research issue.

The goal of this work was the sensory characterization of traditional spontaneously fermented and potentially probiotic green Spanish-style Manzanilla table olives. The study is based on the data obtained from the evaluation of olives from both fermentation systems by 200 consumers. The results are analyzed by ANOVA and multivariate techniques. The study may be useful for predicting the possible response of consumers against the eventual commercialization of potentially probiotic table olives.

## Materials and methods

### Processing

The experiment was carried out with Manzanilla fruits (*Olea Europaea pomiformis*) from the same lot debittered, washed, and brined similarly. In short, it consisted on treating 60 kg olives with 40 L lye (2.2 g NaOH/100 mL) until this reached 2/3 of the flesh (~5 h), followed by an overnight washing to remove the excess of alkali. The debittered fruits were then brined with 40 L 11% NaCl solution, acidified with 0.5 L 10 N HCl. After 2 days, the pH was down corrected to 7.5 units at equilibrium by bubbling CO_2_. Finally, the olives were subjected to two different fermentation systems. One sub-lot was allowed fermenting spontaneously while another one was inoculated with the potentially probiotic bacteria *L. pentosus* TOMC-LAB2 (LAB2), which population size was chosen to reach ≈6 log_10_CFU/mL after addition to the fermenters. The strain had been isolated from wild spontaneous green Spanish-style fermentation processes and was selected because its promising probiotic results according to *in vitro* phenotypic tests (high resistance to gastric and pancreatic digestion, hydrophobicity, auto-aggregation, or capacity of deconjugating bile salts) (15). Their fermentative processes are described elsewhere (31).

### Fermented olive packaging

The fruits from two replicates of the spontaneous and LAB2 inoculated processes were thoroughly washed and packaged in plastic containers (5 kg olives and 2.7 L brine). The characteristics of the packing brine were fixed to reach, after equilibrium in a cold room (7°C), 5.5 g NaCl/100 mL, and 0.6 g lactic acid/100 mL, similar to the usual concentrations of these parameters in the commercial presentations (11). Before being tested by consumers, samples from each replicate of both fermentation methods were withdrawn and analyzed. The study included brine parameters, fruit characteristics, and commercial classification (after tempered at room temperature).

### Physicochemical analyses

The physicochemical characteristics of the cover brines were analyzed according to the methods used routinely in table olive control (11). The olive color was measured using a BYK-Gadner Model 9000 Color View Spectrophotometer (Silver Spring, MD, USA), covering the samples with a box which had a matt black interior. Olive color was expressed as CIE L^*^, a^*^, b^*^ parameters. Color Index (*Ci*) was estimated by the formula: Ci = $\frac{-2^{*}R_{560}\;+\;R_{590}\;+\;4^{*}R_{635}}{3}$, where *R*s are for the reflectance at 560, 590, and 635 nm, respectively (32). Recorded data were the average of 10 olive measurements.

The firmness of the olives was measured on three pitted olives using a Kramer shear compression cell coupled to an Instron Universal Machine (Canton, MA, USA) with a crosshead speed of 200 mm/min. Recorded data were the mean of three olive replicates, expressed as N/100 g flesh.

### Microbiological analyses

The microbiological analyses of brines and olives were performed according to previously described procedures (29, 31, 33). Briefly, appropriate dilutions of the brine samples were plated using a Spiral System model DwScientific (Don Whitley Sci. Ltd., Shipley, UK). The following media were used for the examination of the usual microbiota: VRBD (Crystal-violet Neutral-red bile glucose)-agar (Merck, Darmstadt, Germany) for *Enterobacteriaceae*; MRS (de Man, Rogosa and Sharpe)-agar (oxoid) supplemented with 0.02% (w/v) sodium azide (Sigma, St., Luis, USA) for LAB; and YM (yeast-malt-peptone-glucose)-agar (Difco^TM^, Becton, and Dickison Company, Sparks, MD, USA) supplemented with oxytetracycline and gentamicin sulfate as selective agents, for yeasts. Plates were incubated for 24 h at 30°C for *Enterobacteriaceae* and 48 h at 30°C for yeasts and LAB; Counting was achieved by a CounterMat v.3.10 (IUL, Barcelona, Spain) image analysis system. Results were expressed as log_10_CFU/mL.

### Characterization of the LAB population on olives

Before the sensory evaluation, the biofilms from the packaged olives (of both fermentation methods) were detached, and 20 LAB isolates from the suspension were randomly obtained and coded S, for spontaneous, and P, for probiotic. The lactobacilli were subjected to RAPD-PCR analysis with primer OPL_5_ according to the protocol described by Rossi et al. (34). Their banding profiles (from 100 up to 4,000 bp) were then compared with those of the inoculated strain. For this purpose, PCR products were electrophoresed on a 2% agarose gel and visualized under ultraviolet light by staining with ethidium bromide. The resulting fingerprints were digitally captured and analyzed with the BioNumerics 6.6 software package (Applied Maths, Kortrijk, Belgium). The similarity among the digitalized profiles was calculated using the Pearson product-moment correlation coefficient. The dendrogram was generated using the Unweighted Pair Group Method using the Arithmetic Average (UPGMA) clustering algorithm.

### Olive classification

Before the consumer test, the packaged olives were subjected to classification analysis to assure they had proper commercial quality. The evaluation was conducted in the standardized testing room of the Food Biotechnology Department (IG-CSIC, Sevilla, Spain) under controlled environmental conditions (temperature, humidity and light), using individual booths. The panelists were 12 experienced judges of the Department's staff, habitual consumers of olives with a high level of training due to their participation for decades in the development of the method for the sensory analysis of table olives, issued by the International Olive Council (35, 36). For the test, the evaluation sheet suggested by IOC (36), with an unstructured 10 cm scale, anchored to 1 (no perception) and 11 (extremely intense) was used. In this test, only those attributes used for olive classification according to the IOC methodology (36) were evaluated. Therefore, the assessment in this phase was limited to abnormal fermentation and other defects (e.g., musty, rancid, cooking taste, soapy, metallic, earthy, and winery-vinegary). The marks on the sheet were measured, from the left anchor, with the precision of one decimal place. The analyses were performed in duplicate.

### Consumer test

The consumer test was based on the gustatory (Acid, Salty, and Bitter) and kinesthetic sensations (Hardness, Fibrousness, and Crispness) of the evaluation sheet from the Methods of Sensory Analysis of Table Olives (36). However, the final sheet also included two additional queries: *Overall score* (liking) and *Buying predisposition*. The same 10 cm unstructured scale (anchor rating 1–11) and measurement procedure as in olive classification were applied. The test was performed in one of the most popular Markets in Seville (Mercado de la Encarnación), in a place close to a table olive shop. Only self-reported table olive consumers, at least 2–3 times a month olive consumer, non-diet restrictions, and persons who agree to perform the test were chosen. As the LAB tested were selected from the natural wild microbiota of spontaneous fermentation, no special issues related to health risks were required according to our Ethical Committee. The packaged samples from spontaneously or LAB2 fermented olives, previously classified as Extra/Fancy, were offered to people, under white light, in 150 mL plastic jars labeled with a randomly chosen three-digit code. The fruits were presented as a completely randomized block design. Water was provided to consumers for rinsing their palates between samples, and a 2 min rest was enforced to minimize the carry-over effect. There was no pre-established period for performing each session. The total number of panelists recruited was 200 (105 women and 95 men, with age ranging 20–65 years old).

### Statistical data analysis

Panel results for the olive classification were analyzed according to the procedure established in the Method of Sensory Analysis of Table Olives (35). Data from the consumer assessment were subjected to ANOVA, and *post-hoc* tests with the objective of finding possible differences between fermentation systems within attributes, *Overall score*, and *Buying predisposition*. Also, the data were subjected to Discriminant Analysis (DA). For mapping the samples and visualizing their relationships with the sensory variables, Principal Component Analysis (PCA), Predictive Biplots (PB), and Canonical Variate Analysis (CVA), which determine the position of the points by the two first canonical variates were used. Also, PLS regression was applied to associate sensory attributes with the *Overall score* and *Buying predisposition* and study the relationship between them. The analyses were performed with XLSTAT 2014 (Addinsoft, Paris, France) and the Biplot GUI package which provides a graphical user interface for the construction of, interaction with, and manipulation of biplots in R (37).

## Results and discussion

### Characteristics of the spontaneously fermented and LAB2 inoculated packaged olives

The fermentation process was considered finished after 4 months (the sugars had been exhausted). Then, the physicochemical conditions, quite similar in both fermentation systems, were approximately: pH, 4.0, lactic acid, 15 g/L; NaCl, 55 g/L. Then, the inoculated with LAB-2 (or just probiotic) and the spontaneously processed olives were conditioned and packaged as specified in the Material and Methods section. After equilibrium in the fresh brine used for packaging, the physicochemical and microbiological features of the containers from both fermentations showed similar levels (Table 1). Furthermore, NaCl and titratable acidity concentrations at equilibrium were also close to the presumed levels.

**Table 1:** Average values of the physicochemical characteristics and microbial populations in the packaging brine from spontaneously and LAB2 fermented olives.

**Fermentation method**	**NaCl (g/100mL)**	**pH**	Titratable acidity (g/100mL)^*^	**Combined acidity (Eq/L)**	**Microbial population in brine (log**_**10**_**CFU/mL)**	
					**LAB**	**Yeast**
Probiotic	5.44 (0.02)	3.63 (0.02)	0.69 (0.05)	0.059 (< 0.001)	3.62 (< 0.01)	4.32 (< 0.01)
Spontaneous	5.46 (0.07)	4.00 (0.32)	0.61 (0.04)	0.060 (0.001)	4.23 (1.04)	4.24 (0.16)

Standard error in parenthesis; No statistical difference between processing methods was found within any parameter;

* *expressed as lactic acid; LAB, lactic acid bacteria; Enterobacteriaceae were never found*.

The lack of significance of the differences between the two methods in pH and LAB populations in brine, which were lower in the probiotic than in the spontaneously fermented olives, was due to the high standard errors found for these parameters in the olive packages which olives followed the spontaneous process. Such variability can be associated with the usual diverse evolution of non-controlled replicates which is the biggest obstacle for achieving homogeneous products with the traditional process (11).

The surface color of the packaged olives (Table 2) was also similar, regardless of the fermentation system, with very close levels for CIE L^*^, a^*^, b^*^ parameters and color index. Only the luminance (L^*^) of the probiotic fruits was slightly (non-significantly) higher than the values found in the spontaneously fermented olives, due to their lower pH. L^*^ and b^*^ values of the color parameters were very similar to those found in the fermented product, but those of a^*^ had increased slightly (31). The values of the three parameters were initially lower (about 48, 27, and 1.83, respectively) in green cv. Halkidiki table olives, fermented with potentially probiotic strains, and packaged under modified atmosphere, regardless of the storage temperature (4 or 20°C). But at the end of the product storage, the values of L^*^ and b^*^ were similar to those found in this work (38).

**Table 2:** Average values of surface color, firmness, and microbial load on packaged fruits from spontaneously and LAB2 fermented olives.

**Fermentation method**	**Surface color**				**Firmness (N/100 g pitted olives)**	**Microbial load on the olive surface (log**_**10**_**CFU/olive)**	
	L^*^	a^*^	b^*^	**Color index**		**LAB**	**Yeast**
Probiotic	54.3 (0.3)	2.6 (0.3)	35.9 (0.2)	26.9 (0.41)	1128 (105)	7.59 (0.04)	4.25 (0.76)
Spontaneous	52.9 (0.3)	2.9 (0.3)	35.3 (0.4)	26.2 (0.18)	1237 (132)	6.71 (0.81)	4.67 (< 0.01)

Standard error in parenthesis; No statistical difference between processing methods was found within any parameter;

* *CIE, parameters; LAB, lactic acid bacteria; Enterobacteriaceae were never found*.

Firmness was also statistically the same in spontaneously fermented and probiotic olives (Table 2). However, packaging had sensibly reduced firmness with respect to the fermented product which was about 1,600 and 1,400 N/100 pitted olives for spontaneous and LAB2 fermented olives, respectively (31). Comparison with other similar Greek products is difficult because the different measurements technologies used, although no marked changes with storage time were observed (38).

Finally, LAB and yeast populations in the olive biofilms from both fermentation methods were similar, but the average in probiotic fruits was higher in the LAB and lower in yeast counts. However, the high variability of the LAB in the spontaneous process and yeast in the probiotic olives prevented the differences from being significant. The opposed relationship between LAB and yeast populations in the biofilm on these olives, regardless of the processing method, are comparable to those found in other works (21, 22) or in packaged olives (39). Also, the microbial populations on olives in this work are comparable with those found in Halkidiki cultivar fermented with probiotic *L. pentosus* and *L. plantarum*, packaged under controlled atmosphere and stored at 20°C; however, at 4°C the LAB population increased, but the yeast counts were less affected. To notice that, in this case, there were no differences in behavior among inoculated and spontaneous treated olives and at the end of the storage period, the influence of temperature was reduced but retaining the prevalence of LAB (38).

In any case, according to Verganovi (10), the LAB population in the olive biofilm of the probiotic olives could provide a considerable intake of the putative probiotic LAB2 strain (Table 2). This high value of LAB on the olives supports the suitability of table olives as carriers of potentially probiotic organisms (25–27). When added this characterisitic to the natural composition of olives, which includes mono and polyunsaturated fat (11 and 1.2 g/100 g olive flesh, respectively), dietary fiber (2.6 g/100 g olive flesh), vitamin E (4.6 mg α-tocopherol/100 g olive flesh), phystosterols (2,700 mg/kg olive fat), and the practical absence of cholesterol and sugars (40–43) as well as the numerous properties attributed to the olive polyphenols (anticancer, hemoprotective, anti-inflammatory, antimicrobial, antihypertensive, among others) (44), the table olives may be considered as a potential symbiotic food.

Therefore, according to the previous comments on this work, the physicochemical characteristics (NaCl, pH, titratable acidity, and combined acidity), olive color (L^*^, a^*^, b^*^, and *CI*), firmness, and microbial populations in brines and on olives (LAB and yeasts) of the packaged putative probiotic olives were similar to those from spontaneous fermentation and the differences observed by consumers could only come from the applied fermentations systems (potential probiotic over spontaneous).

### Characterization of the LAB population in the olive biofilm

**Figure 1:**
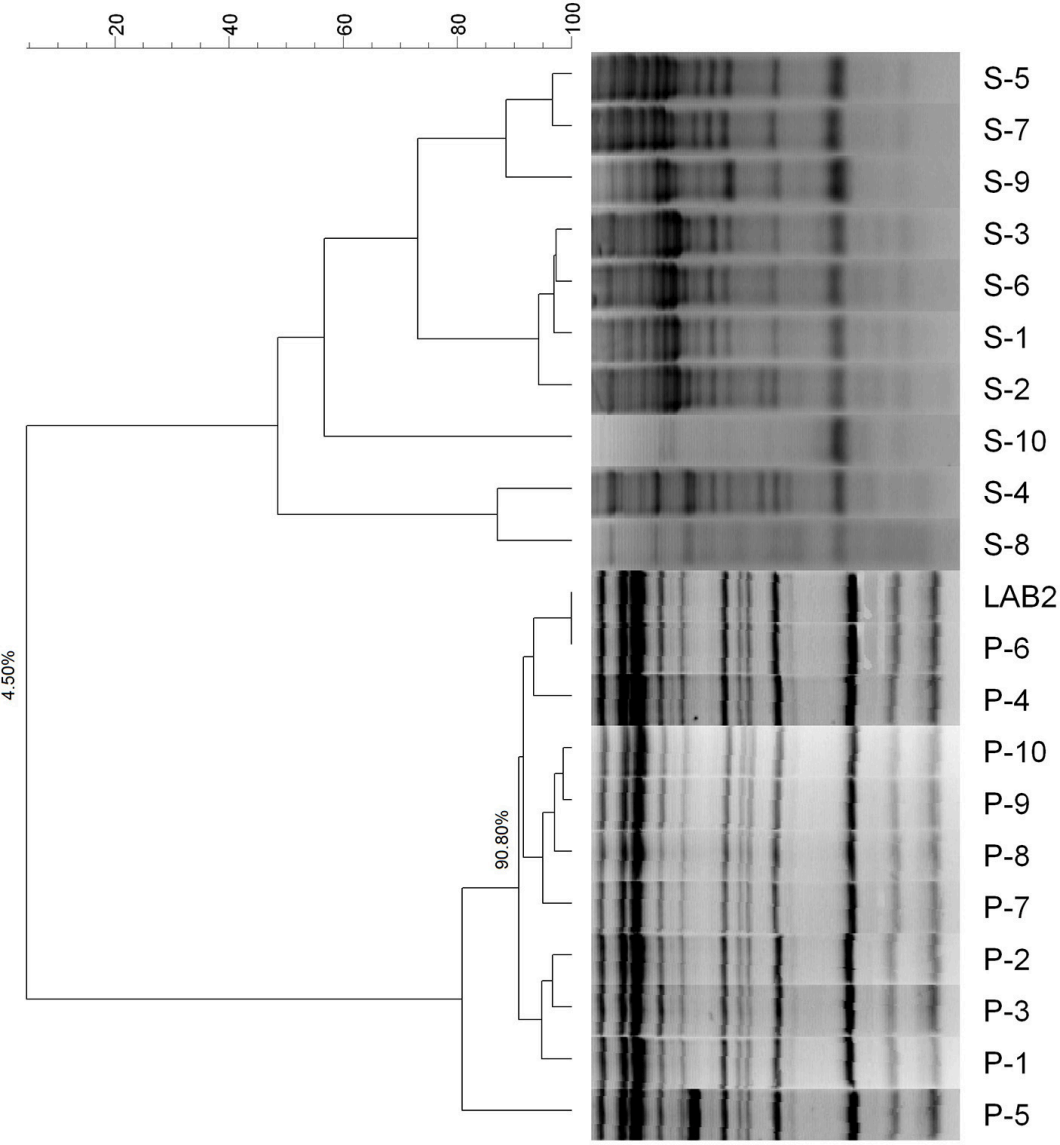
Dendrogram based on RAPD profiles of the LAB isolated from the surface of the packaged spontaneously (S) and LAB2 (P) fermented olives.

The molecular analysis of the 20 LAB strains isolated from the biofilms, both the spontaneous and probiotic fermented packaged olives, showed that the profiles of the LAB population on the fruits from the probiotic olives was 90% similar to that of the LAB2 strain used as starter (Figure 1). On the contrary, the LAB isolates from the spontaneously fermented packaged olives only had 4.5% similarity with LAB2 and, therefore, their strains were completely different. Furthermore, the isolates from the spontaneous fermentation formed six different clusters. As a result, the packaged fruits from the inoculated fermentation were, effectively, carriers of the putative probiotic LAB2 strain used as starter while the olives from the spontaneous process carried only wild strains (with six different genotypes). The consumer test was then performed between real potentially probiotic carriers of LAB2 (inoculated olives) and wild LAB (spontaneous fermentation).

In the already mentioned packaging of cv. Halkidiki under controlled atmosphere, the survival rate of *L. pentosus* B281 was always higher than that of and *L. plantarum* B282, and with time the percentage of both decreased sensibly, and only *L. pentosus* B281 was found after 168 days (38). In Spanish-style Manzanilla olives fermented with putative probiotic LAB, the highest counts (6.2–6.5 log_10_CFU/mL) on olive epidermis were found in fruits stored in glass jars and plastic pouches at 22°C. In general, the correlation between the LAB and yeasts populations were always negative (39). In fortified Manzanilla packaging, the inoculated treatments always had higher populations on the olive surface (4.41–6.77 log_10_CFU/cm^2^) than their respective controls (0.00–4.33 log_10_CFU/cm^2^) and the added strain (*L. pentosus* TOMC-LAB2) was recovered at the end of the shelf life (200 days) at frequencies ranging 53–100% (45).

### Packaged olive classification

The objective of this test was checking that the olives from the two fermentation methods (spontaneous and probiotic) had appropriate commercial quality. Then, it was considered as a first step in the evaluation of the packaged fruits. After analyzing the scores assigned by the panel to abnormal fermentation and other defects, the average values (data not shown) were very close to 1.0. Then, the levels obtained were far below the set upper limit (3.0) for considering any sign of spoilage according to the Sensory Analysis of Table Olives (36) and, subsequently, the olives from both fermentation methods were classified as Extra (or Fancy). Therefore, both the spontaneously fermented and potentially probiotic packaged olives had the highest commercial quality and were appropriate for consumer evaluation.

### Results of the consumer test

#### Sensory scores of spontaneously fermented and potentially probiotic packaged olives

The average scores for the gustatory and kinesthetic sensations as well as for *Overall score* and *Buying predisposition* were similar and did not lead to any significant difference (*p* < 0.05) between the spontaneously fermented and potential probiotic packaged olives (Table 3). Also, the proportions of consumers who assigned a higher *Overall score* to the potentially probiotic and to the spontaneous olives (or vice versa) were statistically the same according to the χ^2^-test.

**Table 3:** Average values of sensory attributes from the consumer test and *Overall score* and *Buying predisposition*, according to olive processing method.

**Sensory variable**	**Average score**		**Statistical comparison**		**Preference**		**Preference test**	
	**Probiotic**	**Spontaneous**	*F*-value^*^	***p*-value**	**Probiotic**	**Spontaneous**	***X*^2^**	***p*-value**
Appearance	9.05 (1.55)	8.99 (1.54)	0.1661	0.68	94	106	0.0036	0.0000
Odor	7.87 (2.08)	8.06 (1.88)	0.9691	0.32	92	102	0.0064	0.0000
Acid	5.27 (2.51)	5.06 (2.50)	0.7179	0.40	110	90	0.0100	0.0000
Bitter	3.21 (2.29)	3.18 (2.24)	0.0127	0.91	111	89	0.0100	0.0000
Salty	7.31 (1.91)	7.13 (1.93)	0.6968	0.40	105	95	0.0025	0.0000
Hardness	4.70 (1.92)	4.56 (1.89)	0.5445	0.46	102	98	0.0004	0.0000
Fibrousness	3.97 (2.24)	3.94 (2.21)	0.0356	0.85	99	101	0.0004	0.0000
Crispness	3.77 (2.12)	3.78 (2.23)	0.0033	0.95	94	109	0.0036	0.0000
*Overall score*	7.96 (1.73)	8.20 (1.56)	2.2855	0.13	92	108	0.0064	0.0004
*Buying predisposition*	7.10 (2.61)	7.33 (2.52)	0.7540	0.39	77^**^	101^**^	0.0196	0.0004

Preference, highest values in the Overall score, is also included. Standard deviation in parenthesis;

* F values for 1 and 398 fd;

** *same score, 22*.

The cv. Halkidiki fermented as Spanish-style table olives with *L. pentosus* B281, *L. plantarum* B282 or subjected to the spontaneous process were also sensory analyzed after packaging in modified atmosphere. At the beginning of the storage, the inoculated samples were preferred (higher acceptability index scores) over those from the traditional process; furthermore, those treated with *L. pentosus* B281 were chosen preferently. However, storage reduced acceptability, although retaining the preferences for the inoculate olives. After 12 months storage period, those olives which followed the spontaneous process were almost unacceptable while *L. plantarum* B282 retained acceptability index similar to those stored for 6 months and was preferred to those fermented with *L. pentosus* B281 (38). After packaging, the cv. Manzanilla fermented with diverse strains of *L. pentosus* showed, for all the attributes evaluated (36), levels around the score center (5.5–6.4) while the fruits from the spontaneous processed developed a clear abnormal fermentation, circumstance that reinforces the convenience of inoculation as a way of the safety initiation of green Spanish-style fermentation (29). Randazzo et al. (46), studied the sensory effect of using probiotic cultures as starters in non-lye treated Giarrafa and Grossa di Spagna and results were significantly cultivar-dependent. In heat-shocked olives, the salty scores were strongly associated with brine concentration but received similar acidity punctuation, except control (lowest) at 8% NaCl. Regarding bitterness, the inoculated process had high levels at 8% NaCl but the control and inoculated with *L. pentosus* were perceived as more bitter when processed at 10% NaCl. The inoculated olives (*L. pentosus and L. plantarum)* at 8% NaCl were the preferred olives, followed by those fermented with *L. plantarum* at 10% NaCl (24).

However, the tendencies observed in this work regarding several attributes like Acid (possibly due to the slightly lower pH of the probiotic packaged olives), Bitter, Salty (also possibly related to the pH level), and Crispness should not be underestimated. They may indicate the presence of some subtle trends in consumer preferences whose in-depth study could be of interest.

#### Multivariate analysis of the scores assigned by consumers

There were many significant (*p* < 0.05) correlations among the attribute scores. For example, Appearance vs. Odor (0.462), Bitter (−0.270), Fibrousness (−0.221), *Overall score* (0.265), and *Buying predisposition* (0.269); Acid vs. Bitter (0.369) and Hardness (0.355); Bitter vs. Fibrousness (0.360), and Crispness (0.455); or *Overall score* vs. *Buying predisposition* (0.688). Therefore, the data were appropriate for multivariate exploration.

**Figure 2:**
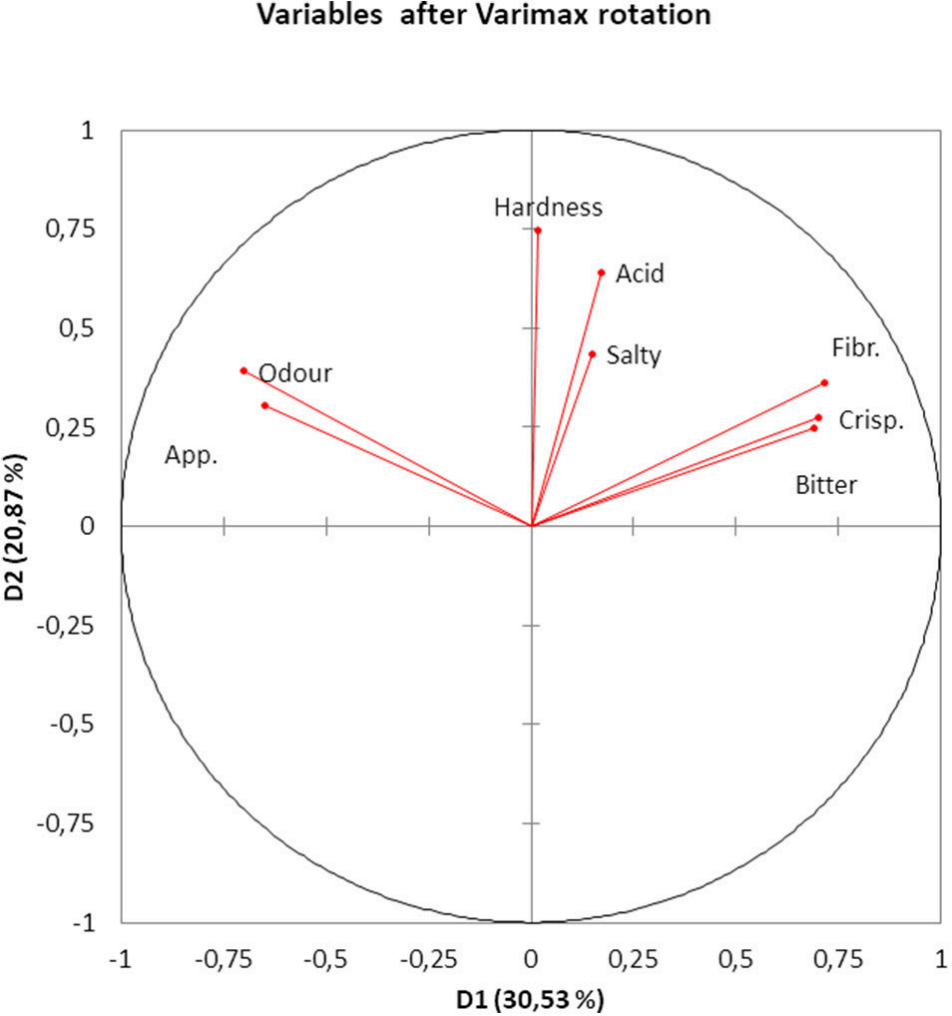
PCA analysis of the result from the consumer test, based on all responses. Projection of variables onto the first two Principal Components. App., Appearance; Fibr., Fibrousness; Crisp., Crispness.

The PCA of the sensory variables extracted four eigenvalues higher than 1 which accounted for ~64.39% of the total variance. Their projection onto the plane of the first two Dimensions (D1 and D2) (~51% of variance), after varimax rotation, showed that Appearance and Odor were closely related but negatively linked to D1 (Figure 2). Crispness, Fibrousness, and Bitter were also closely related and positively correlated to D1 (Figure 2). D1 could then be associated with maturation degree. In fact, the opposed relationship between the previous two groups of attributes is reasonable since the less mature (higher Bitterness, Fibrousness, and Crispness) the more difficult is obtaining the typical Spanish-style characteristics (Odor and Appearance) after fermentation. D2 was linked to Hardness which, in turn, was hardly associated to Odor and Appearance or Fibrousness, Crispness and Bitter because of the close to 90° angle (cosine = 0) between the first and the last two groups (Figure 2). Finally, the vectors of Acid and Salty (mainly), although following similar directions (linked to some correlation), were relatively short, indicating that they might be not well represented by the first two Dimensions; in fact, Acid was closely related to D3 (correlation, 0.6379) and Salty to D4 (0.796). The graph was then useful to show the association among variables in an intuitive way and revealed that the number of variables evaluated in the Sensory Evaluation of Table Olives (36) could be reduced and the analysis simplified.

In case of Halkidiki cultivar Spanish-style table olives, clustering was mainly achieved regarding storage time, with the greatest distance between olives stored for 168 days and the rest with slight differences between 0 and 370 days storage. By using PCA, three segregated groups were noticed, with time being also the most influential parameter. Also, olives samples after 6 months storage were strongly correlated with L^*^ while those with 12 months storage were linked to b^*^ values. Besides, the just packaged olives were associated with acidity, a_W_, and a^*^ values and the olives at the end of storage with pH (38).

Due to the high number of consumers and their random distribution close to and around the origin (without any clear segregation between potential spontaneously fermented and probiotic olives), their relationships in the plane of the first two Dimensions were difficult to interpret. Furthermore, the DA led to a correct overall classification of around 50%, similar to that expected just by random assignation to groups. However, a more in-depth study of the tendencies mentioned above could still be of interest.

#### Mapping the sensory tendencies by biplot

One approach to this study was accomplished by condensing the answers of consumers into a reduced number of data. In this way, the number of cases would be manageable without losing information. To this aim, the initial 200 consumers were randomly assigned to 8 groups of 25 consumers each, and their sensory variable averages estimated. The values obtained (eight averages per fermentation method), representing the population of answers, were now a manageable number of cases, with an easy visualization on any graph and, particularly, on the biplots.

**Figure 3:**
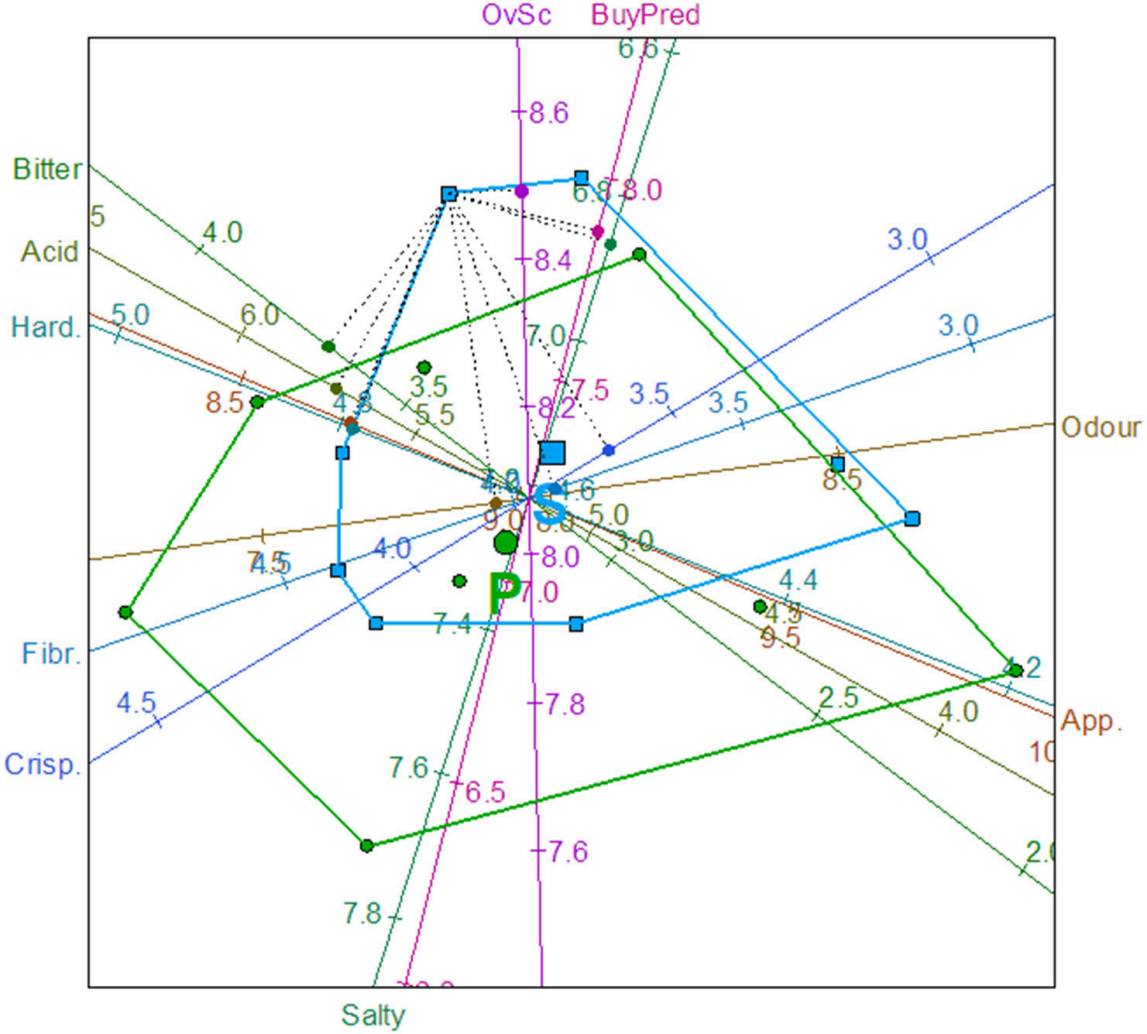
PCA Biplot of the average over 25 consumers according to sensory variables (App., Appearance; Hard., Hardness; Fibr., Fibrousness; Crisp., Crispness), *Overall score* (OvSc), and *Buying predisposition* (Buypre). Convex hulls for spontaneously (S) and potentially probiotic (P) fermented olives. Predictions for one average, are also shown.

Usually, a biplot simultaneously provides information on both the samples and the variables of a data matrix in two and, if necessary, three dimensions. A recent improvement consists of incorporating information on the original variables by linear or non-linear axes. Implementation of the goodness of fit measurements, convex hulls, and classification regions markedly assist in the interpretation of results (37). In this work, the PCA biplot with predictive axes was applied to the data matrix formed with the eight averages of each sensory attribute and their respective *Overall score* and *Buying predisposition*. Apart from the relationships among variables, in the biplot obtained (Figure 3) because of the use of calibrated axes, it is possible to read the predicted values (and their errors) of each point (average of 25 answers) by projecting perpendicular lines onto the respective axes. For example, the predicted (average) values for the left upper point in the second quadrant would be: Appearance, 8.7 (with a relative error of 8.3%); Odor, 7.90 (14.6%); Acid 5.73 (1.1%); Bitter, 3.69 (3.9%); Salty, 6.87 (11.0%); Hardness, 4.79 (5.9%); Fibrousness, 3.91 (15%); Crispness, 3.62 (17.6%); *Overall score*, 8.49 (12.7%), and *Buying predisposition*, 7.87 (11.6%). The predictions of the biplot were then fairly good for some variables (e.g., Acid and Bitter) while were relatively less precise for others (e.g., Fibrousness and Crispness). However, the most relevant information from this graph (Figure 3) is the disposition of the averages. The centroids of the convex hulls (with the corresponding symbols in a bigger size) were near and the regions associated with spontaneous (S) and probiotic (P) olive fermentations overlapped. Nevertheless, the displacement observed between their convex hulls indicates that there was a tendency for scoring the probiotic olives to the bottom of the graph, although the biplot was not efficient enough for disclosing the possible small differences between the two fermentation methods.

**Figure 4:**
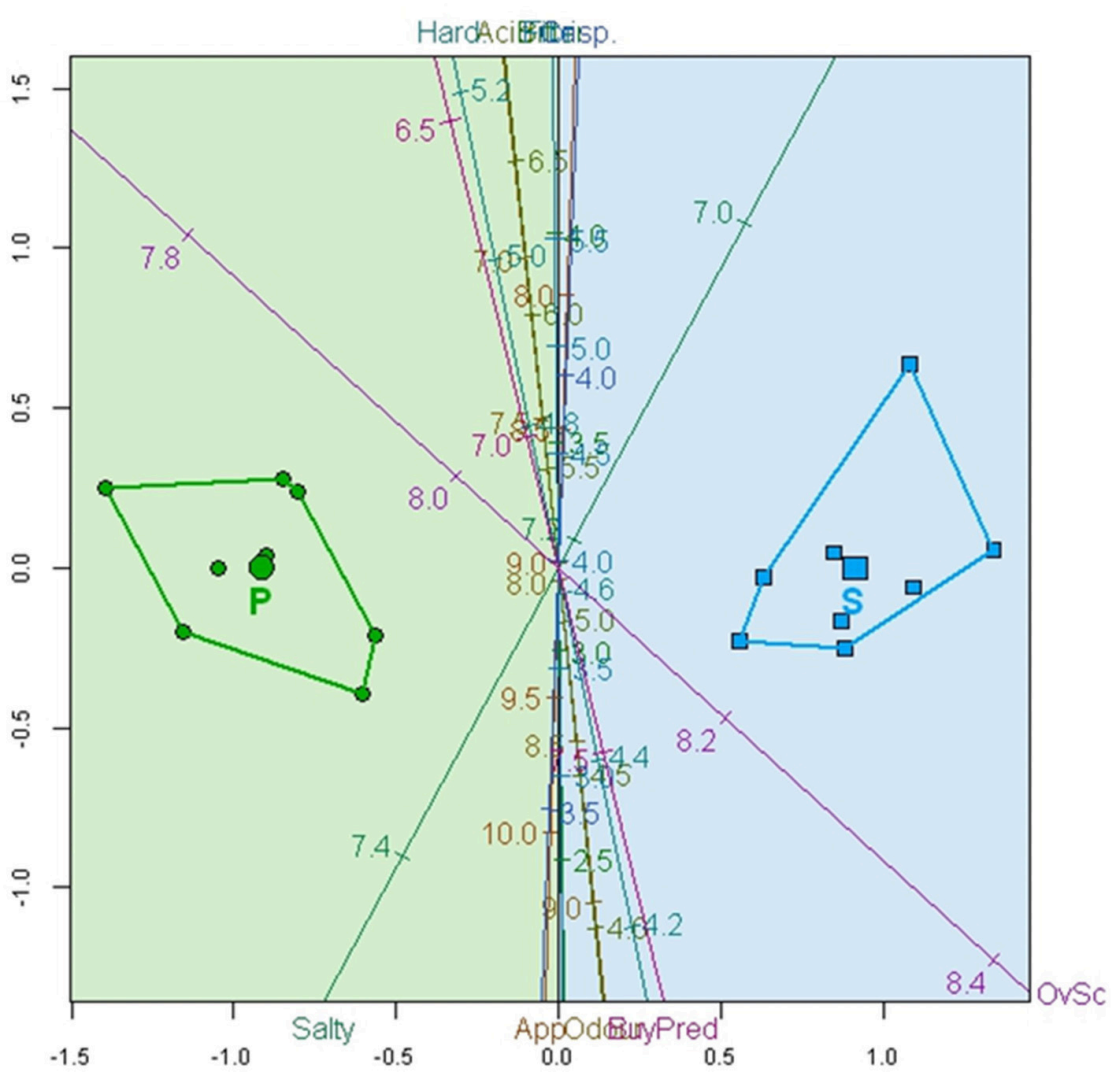
Biplot based on CVA analysis of the average over 25 consumers according to sensory variables (App., Appearance; Hard., Hardness; Fibr., Fibrousness; Crisp., Crispness), *Overall score* (OvSc), and *Buying predisposition* (Buypre). Convex hulls for spontaneously (S) and potentially probiotic (P) fermented olives are also included.

However, a biplot based on the CVA, whose axes are estimated as linear combinations of the original variables that maximally separate the group means, could be even more convenient. With the CVA predictive biplot, the potentially probiotic (on the left) and spontaneously fermented (on the right) olives were segregated (Figure 4). Most of the calibrated variable axes are close to the vertical canonical axis and, as a result, had a limited segregating capacity; however, Salty and *Overall score*, which form a close to 90° axes, can be considered responsible for the segregation. The biplot also associated the region on the left with the probiotic olives and that on the right with the spontaneous method. Probiotic fermented olives are then characterized by 7.2–7.4 Salty scores and 7.8–8.1 *Overall scores* while spontaneously fermented olives have lower than 7.2 Salty score and higher than 8.1 *Overall score*.

#### Relationship between sensory attributes with the overall score and buying predisposition

**Figure 5:**
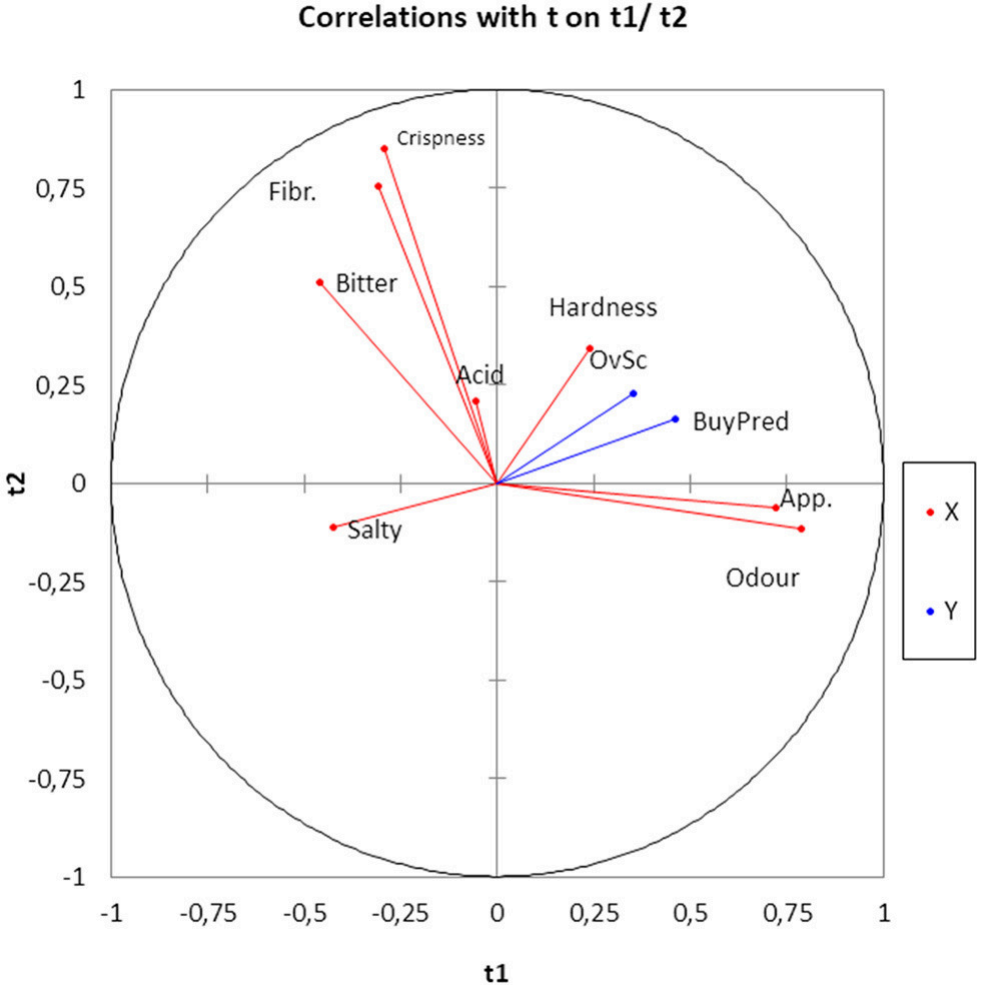
Sensory variables, *Overall score* and *Buying predisposition* projection onto the t1 and t2 plane as the deduced by PLS analysis. Fibr, Fibrousness; OvSC, *Overall score*, BuyPred, *Buying predisposition;* App, Appearance.

Using the sensory attributes as independent variables and the *Overall score* and *Buying predisposition* as a dependent (responses), the relationships between them in the whole matrix of data were quantified by PLS. The application of this statistical tool is rather appropriate since it is particularly useful in case of correlations among predictor variables. Quality indices of the regression for the model with two components (Q^2^ accumulated, 0.155; R^2^Y accum, 0.206; and R^2^X, 0.440) showed a low proportion of the total, Y and X variance explanation. Apparently, some of the motivations of consumers for scoring or buying table olives might not be included in the official evaluation sheet for table olives (36). Subsequently, the analyzed sensory variables had a limited prediction power. The most important variables in the two-component model were Odor and Appearance, in agreement with their proximity to the component t1 and t2 plane (Figure 5). Apart from t1, *Overall score and Buying predisposition* were also clearly associated with Hardness, Appearance, and Odor while were negatively related to Salty (Figure 5). Mathematically, the equation of *Overall score* and *Buying predisposition* as a function of the sensory attributes were deduced and their standardized coefficients, standard errors, and confidence limits estimated (Table 4), with the standardized coefficients being a measurement of their contributions to the responses, regardless of their physical levels. Most of the coefficients were significant and, therefore, predictions can still represent an attractive tool to disclose consumers' attitude. The equations for *Overall score* and *Buying predisposition* had similar structures (coefficients), in agreement with their high correlation (Figures 4, 5); that is, the same attributes have significant contributions (positively and negatively) to both *Overall score* and *Buying predisposition* (Table 4). Appearance, Odor, Hardness, and Crispness (positive contributions) improve consumer perception while Salty has a substantial adverse impact.

**Table 4:** Standardized coefficients for the *Overall score* and *Buying predisposition* estimated by PLS-R as a function of the sensory attributes.

**Sensory variable**	***Overall score***				***Buying predisposition***			
	**Coeff**.	**SE**	**LCL**	**UCL**	**Coeff**.	**SE**	**LCL**	**UCL**
Appearance	0.200^*^	0.047	0.108	0.293	0.242^*^	0.040	0.164	0.319
Odor	0.194^*^	0.052	0.092	0.295	0.241^*^	0.036	0.171	0.312
Acid	−0.003	0.065	−0.131	0.126	−0.005	0.077	−0.156	0.146
Bitter	0.000	0.037	−0.073	0.073	−0.032	0.042	−0.115	0.050
Salty	−0.228^*^	0.058	−0.343	−0.114	−0.250^*^	0.069	−0.385	−0.114
Hardness	0.116^*^	0.045	0.028	0.204	0.128^*^	0.053	0.024	0.232
Fibrousness	0.082	0.064	−0.044	0.208	0.054	0.056	−0.056	0.164
Crispness	0.159^*^	0.048	0.065	0.252	0.128^*^	0.053	0.025	0.232

SE, standard error; LCL and UCL, Low and upper confidence interval (for p ≤ 0.05); Coeff., coefficients for independent variables;

* *significant at p ≤ 0.05*.

The equations (in physical values) were the following:

*Overall score* = 5.06 + 0.22^*^App. + 0,17^*^Odor − 1.73E − 03^*^Acid − 5.26E − 05^*^Bitter − 0.20^*^Salty + 0.10^*^Hard + 6.17E − 02^*^Fibr. + 0.12^*^Crisp.

*Buying predisposition* = 2.01 + 0.41^*^App. + 0.32^*^Odor − 4.97E − 03^*^Acid − 3.66E − 02^*^Bitter − 0.33^*^Salty + 0.17^*^Hard + 6.25E − 02^*^Fibr. + 0.15^*^Crisp.

**Figure 6:**
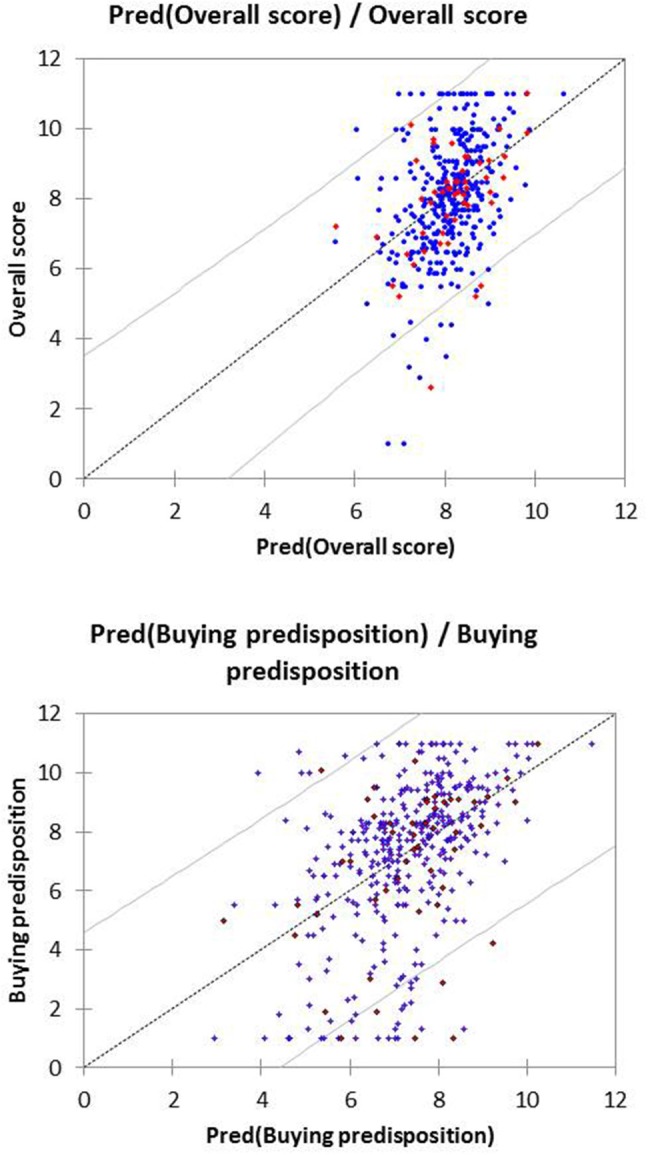
Prediction and their confidence limits (*p* ≤ 0.05) from the PLS analysis of the *Overall score* and *Buying predisposition* as a function of the sensory variables. Only 25 cases (active and validation) randomly selected are represented, as examples.

The performance of these equations was checked by representing the scores assigned by the consumer to these variables vs. the predicted values (obtained from the equations). To facilitate the observation, only 25 cases of those used for the model development and validation, randomly selected, are included in the graph (Figure 6). Only a reduced number of cases are outside the Confidence Limits (CL) (*p* ≤ 0.05), regardless of the response.

According to the equations, for increasing table olive valorization by consumers, Appearance, Odor (appropriate fermentation), Hardness, and Crispness should be improved (mainly the first two attributes). On the contrary, the Salty perception should be reduced because of its sensible negative influence on the *Overall score* and *Buying predisposition*. The significant negative contribution of Salty (the highest) to *Overall score* and *Buying predisposition* is in agreement with the consumer concern for the association of salt with cardiovascular diseases (47) and the firm determination of consumers (and authorities) to reduce its intake (48–50). Since the consumers' scores regarding salt reflected was not influenced by any previous questing on this issue, they reflected their current spontaneous attitude. Therefore, reducing the Salty perception, and the clarification of the relationship among this attribute with pH and Acid (frequently not well differentiated), should then be a priority for a better understanding of the consumers' attitude not only toward probiotic but to table olives in general. Furthermore, due to the favorable influence of low Salty scores on table olive appreciation by consumers, a possible reduction in the salt level of the potentially probiotic olives could be a very straightforward and efficient strategy to facilitate their commercialization. However, the motivation of consumers is by no means simple. In a survey with the objective of studying the motivation of the Albanian population for the consumption of olives, including their origin, Zhllima et al. (30) chosen a limited number of questions namely origin (imported/local), color (green/dark brown), type (plain, pitted, and stuffed) as well as the price they were willing to pay. Using Cojoint Choice Experiment and Latent Class Analysis, the authors were able to segment the demand into four main classes, associating Class 1, mainly with origin; Class 2, with color; Class 3, with price; and Class 4, with price and type. Therefore, many another aspects should also be borne in mind when commercializing table olives. In any case, due to the favorable influence of low Salty scores on table olive appreciation by consumers, a possible reduction in the salt level of the potentially probiotic olives could be a very straightforward and convenient strategy to facilitate their introduction in the market.

## Conclusion

Overall, green Spanish-style potentially probiotic olives are perceived by consumers as similar to the traditional product. In fact, ANOVA and DA were unable to disclose any differences among them. However, the application of Predictive Biplot based on CVA revealed consumer trends based mainly on the Salty perception and *Overall score*. The sensory attributes with favorable influence on the *Overall score* and the *Buying predisposition* were Appearance, Odor, and Crispness (in a lower proportion); on the contrary, Salty had a marked adverse effect. Therefore, an association of probiotic olives with low Salty perception could facilitate its commercialization.

## Author contributions

AL-L, JM-B, PG-G, and AG-F performed the consumers' test. FR-G characterized the physicochemical and microbiological characteristics of the products. AL-L and AG-G designed and supervised the experiment, analyzed and interpreted the data, and drafted the manuscript. All authors approved the final version of the paper.

### Conflict of interest statement

The authors declare that the research was conducted in the absence of any commercial or financial relationships that could be construed as a potential conflict of interest.
